# Lead and δ-Aminolevulinic Acid Dehydratase Polymorphism: Where Does It Lead? A Meta-Analysis

**DOI:** 10.1289/ehp.9448

**Published:** 2006-09-15

**Authors:** Franco Scinicariello, H. Edward Murray, Daphne B. Moffett, Henry G. Abadin, Mary J. Sexton, Bruce A. Fowler

**Affiliations:** 1 Division of Toxicology and Environmental Medicine, Agency for Toxic Substances and Disease Registry, Centers of Disease Control and Prevention, Atlanta, Georgia, USA; 2 Epidemiology Consultant, Atlanta, Georgia, USA

**Keywords:** *ALAD* polymorphism, lead, meta-analysis

## Abstract

**Background:**

Lead poisoning affects many organs in the body. Lead inhibits δ-aminolevulinic acid dehydratase (ALAD), an enzyme with two co-dominantly expressed alleles, *ALAD1* and *ALAD2*.

**Objective:**

Our meta-analysis studied the effects of the *ALAD* polymorphism on *a*) blood and bone lead levels and *b*) indicators of target organ toxicity.

**Data source:**

We included studies reporting one or more of the following by individuals with genotypes *ALAD1-1* and *ALAD1-2/2-2*: blood lead level (BLL), tibia or trabecular lead level, zinc protoporphyrin (ZPP), hemoglobin, serum creatinine, blood urea nitrogen (BUN), dimercaptosuccinic acid–chelatable lead, or blood pressure.

**Data extraction:**

Sample sizes, means, and standard deviations were extracted for the genotype groups.

**Data synthesis:**

There was a statistically significant association between *ALAD2* carriers and higher BLL in lead-exposed workers (weighted mean differences of 1.93 μg/dL). There was no association with *ALAD* carrier status among environmentally exposed adults with BLLs < 10 μg/dL. *ALAD2* carriers were potentially protected against adverse hemapoietic effects (ZPP and hemoglobin levels), perhaps because of decreased lead bioavailability to heme pathway enzymes.

**Conclusion:**

Carriers of the *ALAD2* allele had higher BLLs than those who were *ALAD1* homozygous and higher hemoglobin and lower ZPP, and the latter seems to be inversely related to BLL. Effects on other organs were not well delineated, partly because of the small number of subjects studied and potential modifications caused by other proteins in target tissues or by other polymorphic genes.

Lead poisoning is a complex disorder affecting many organs in the body, including developing red blood cells, the kidneys, and the nervous system. Young children are most susceptible to the toxic effects of lead. Major concerns are the cognitive and neurobehavioral deficits resulting from lead exposure levels that were previously considered safe. High levels of exposure can cause encephalopathy and death [Agency for Toxic Substances and Disease Registry (ATSDR) 1999].

Lead deposition in the body consists of three major pools: blood, bone, and soft tissues (Rabinowitz et al. 1976). The blood pool accounts for only 2% of the total body burden, unless there is an acute exposure, but is a rapidly exchangeable component. The bone pool contains > 95% of the total body burden, where it may be mobilized and contribute to the blood lead level (BLL) in previously exposed persons. Differences in lead accumulation in various bone types have been reported. Tibia concentrations differ from those observed in the patella. The cortical bone of the tibia represents a long-term storage depot with an elimination half-life for lead in excess of a decade. In contrast, the more dynamic trabecular bone of the patella exhibits a shorter half-life (Rabinowitz et al. 1976).The remainder of the total body load is distributed in an intermediate pool of soft tissues, skin, and muscle. Elimination half-lives for lead are estimated at 30–40 days in blood and up to 20 years or longer in bone (Marcus 1985a, 1985b). Lead is eliminated mainly in the urine.

Lead is a potent inhibitor of δ-aminolevulinic acid dehydratase (ALAD), copropor-phyrinogen oxidase, and ferrochelatase, enzymes that catalyze the second, sixth, and final steps, respectively, in the biosynthesis of heme (Onalaja and Claudio 2000; Warren et al. 1998). Because the metal has the greatest effect on ALAD, measurement of ALAD activity can be used as a marker of effect of lead exposure (Chisolm et al. 1985). ALAD, an octameric zinc-containing enzyme, catalyzes the condensation of two molecules of 5-aminolevulinic acid (ALA) into one molecule of monopyrrole porphobilinogen (PBG). Inhibition of ALAD activity produces increased urinary excretion of ALA (Warren et al. 1998).

Lead displaces zinc from the enzyme’s active site, and the inactivation of ALAD has been implicated in the pathogenesis of lead poisoning. The resulting accumulation of its substrate, ALA, has been shown to have a neuropathogenic effect, probably by acting as a γ-aminobutyric acid (GABA) receptor agonist in the nervous system (Brennan and Cantrill 1979).

Human ALAD, encoded by a single gene localized to the chromosome 9q34 region, is a polymorphic enzyme with two alleles, *ALAD1* and *ALAD2* [Single Nucleotide Polymorphism database (dbSNP) ID: rs1800435; http://www.ncbi.nlm.nih.gov/SNP/index.html], which are co-dominantly expressed (Battistuzzi et al. 1981). The difference between the two alleles lies in a single G→C transversion mutation of nucleotide 177 in *ALAD2*; the allozyme resulting from the *ALAD2* allele contains the substitution of a neutral asparagine for a positively charged lysine at residue 59 (Wetmur et al. 1991b). Three differently charge allozymes, ALAD1-1, 1-2, and 2-2, result from the expression of the *ALAD1* and *ALAD2* genes. In several white populations, the frequencies of the *ALAD1* and *ALAD2* genes have been estimated to be 0.9 and 0.1, respectively. Asian and African populations have lower frequencies of the *ALAD2* allele (Kelada et al. 2001).

Several epidemiologic studies have attempted to correlate the *ALAD* allelic variations with a differential susceptibility to lead poisoning. The biologic plausibility for a differential role of the two alleles lies in the fact that the lysine substitution at residue 59 changes the electrical charge of the enzyme (Battistuzzi et al. 1981); the more electronegative ALAD2 enzyme may thus have a higher affinity/stability for the lead cation than ALAD1 (Wetmur et al. 1991b). This could result in an alteration of lead toxicokinetics and susceptibility to lead toxicity. The first studies comparing BLL and *ALAD* polymorphism were conducted on a chronically exposed population of 202 male lead workers in a German factory (Ziemsen et al. 1986), and an environmentally exposed population of 1,051 children with elevated free erythrocyte protoporphyrin (Astrin et al. 1987). These studies showed that individuals carrying one or two copies of the *ALAD2* allele exhibited higher BLLs than homozygous individuals with only the *ALAD1* allele. These findings led to the suggestion that *ALAD2* may be a determinant for increased susceptibility to lead toxicity (Wetmur et al. 1991a). However, some studies have reported either no difference among individuals homozygous for *ALAD1* relative to individuals carrying the *ALAD2* allele, or the differences among the two groups were not statistically significant. The extreme variability in the published data is due to several factors: relatively small numbers of subjects, different frequencies of the *ALAD2* allele in various populations, and different levels of lead exposure as determined by BLLs in the populations studied. We used a series of meta-analyses to quantify the effects of this genetic polymorphism and to understand lead toxicokinetics.

## Methods

### Study selection

MEDLINE (National Library of Medicine 2006) and Web of Science (Thomson Scientific 2006) databases were searched to January 2006 for English-language publications of observational studies. The citations in the articles identified were also searched to find other potentially eligible studies. Common text words and Medical Subject Headings (MeSH) related to lead poisoning, gene polymorphism, and ALAD were used. No attempt was made to contact the authors of any of the articles, except to resolve discrepancies in the reported values.

We required that two *a priori* criteria be met for inclusion in the meta-analysis: *a*) sample sizes, means, and SDs were either reported or could be determined for the *ALAD1-1* and *ALAD1-2/2-2* genotypes; and *b*) combined with one or more of the following measures—BLL, tibia lead level, trabecular (patella or calcaneus) lead level, zinc protoporphyrin (ZPP), hemoglobin, serum creatinine, dimercaptosuccinic acid–chelatable lead, and systolic or diastolic blood pressure. When multiple studies used the same cohort of subjects, the first publication that reported the values of the variables of interest was included.

### Data extraction

Sample sizes, means, and SDs according to genotype (homozygous *ALAD1-1* and *ALAD2-2* and heterozygous *ALAD1-2*) were extracted independently by two authors (F.S and D.M.). Wu et al. (2004) reported data in groups of workers subjected to high and low lead exposures. We mathematically combined the data of the two groups to extract the means for all exposed workers according to the genotype. The pooled estimate of the variance from two independent samples was used to extract the SDs according to genotype. Therefore, only one effect size was entered in the model. The data from each study were entered twice to minimize data-entry errors.

### Statistical analysis

The data were analyzed using Stata software version 7 (StataCorp., College Station, TX, USA). In each study the size of the effect was calculated by the difference between the means of the *ALAD1-2/2-2* and the *ALAD1-1* groups. Each mean difference was weighted according to the inverse of its variance, and the average was taken [weighted mean difference (WMD)]. To combine data from studies in which the same outcome was measured by different scales (serum creatinine), or when the outcome value was measured by different methods (bone lead, ZPP), the mean difference was standardized by dividing by the within-group SD; the results were then weighted and the average, or standardized mean difference (SMD), taken. The WMD or SMD in each study was pooled using a random-effects model. Results are given with 95% confidence intervals (CIs). Between-study heterogeneity in the results of the studies was assessed using a chi-square test and the *I*^2^ measure of inconsistency. Significant heterogeneity was defined as a chi-square test *p-*value < 0.1. *I*^2^ takes values between 0% and 100% with higher values denoting greater degree of heterogeneity (*I*^2^ = 0–25%: no heterogeneity; *I*^2^ = 25–50%: moderate heterogeneity; *I*^2^ = 50–75%: large heterogeneity; *I*^2^ = 75–100%: extreme heterogeneity) (Higgins et al. 2003). Furthermore, to examine between-study heterogeneity, we used *a priori* stratified analyses including the study design (occupational and environmental studies) and age status (children and adults) and presence of Hardy-Weinberg equilibrium (HWE). Publication bias was assessed using the methods proposed by Begg and Mazumdar(1994) and by Egger et al. (1997). All *p-*values are two-tailed.

## Results

**Figure 1:**
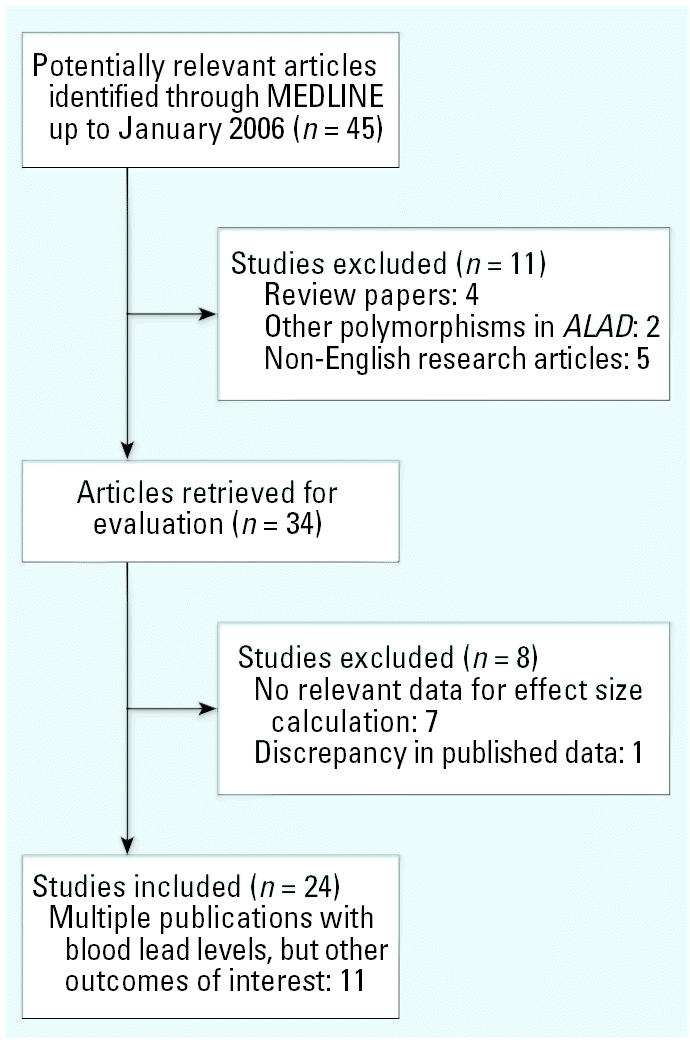
Flow chart of study selection.

The search procedure yielded 45 references that were retrieved for additional information (Figure 1). We initially excluded 4 review papers, 5 non-English research articles, and 2 articles that reported data on different variant of the *ALAD2* polymorphism. Of the remaining 34 articles, 7 did not have relevant data for effect size calculation. Moreover, the corresponding author of a study of environmentally exposed children (Shen et al. 2001) was contacted twice by monthly e-mail to resolve some discrepancy in their reported study. Three months after failing to receive an answer, we decided to exclude the study. Therefore, 24 studies were included in the meta-analysis (Alexander et al. 1998; Astrin et al. 1987; Duydu and Suzen 2003; Fleming et al. 1998; Hsieh et al. 2000; Hu et al. 2001; Kim et al. 2004; Lee BK et al. 2001; Lee SS et al. 2001; Perez-Bravo et al. 2004; Sakai et al. 2000; Schwartz et al. 1995, 1997a, 1997b, 2000; Sithisarankul et al.1997; Smith et al. 1995; Suzen et al. 2003; Theppeang et al. 2004; Weaver et al. 2003; Wetmur et al. 1991a; Wu et al. 2003, 2004; Ziemsen et al. 1986), and of these, 11 were multiple publications that often had other different outcomes of interest. When we found a discrepancy in the reported studies, the authors were contacted and the corrected data were used. Table 1 characterizes the studies that did meet criteria for inclusion.

### ALAD *polymorphism and blood lead level.*

Nine occupational studies (Alexander et al. 1998; Fleming et al. 1998; Kim et al. 2004; Sakai et al. 2000; Schwartz et al. 1995, 2000; Suzen et al. 2003; Wetmur et al. 1991a; Wu et al. 2004) were included in our analysis, and 5 environmental exposure studies of which 3 were conducted among adults (Smith et al. 1995; Hsieh et al. 2000; Wu et al. 2003) and 2 among children (Perez-Bravo et al. 2004; Wetmur et al. 1991a). Thus, a total of 14 studies were included in our analysis of blood lead level and *ALAD* polymorphism. Each of the studies was rechecked for HWE. We did not find HWE in the study by Wetmur et al. (1991a) that presented separate data on previously reported studies of occupational exposure in adults (Ziemsen et al. 1986) and environmental exposure in children (Astrin et al. 1987). The absence of HWE is most likely because of ethnicities of the populations: the occupational study comprised workers of German and Turkish origins (Ziemsen et al. 1986), whereas the study of children included whites, blacks, Hispanics, and Asians (Astrin et al. 1987). Table 2 shows the frequency of the *ALAD* polymorphism and the status of the HWE in the studies analyzed.

There is evidence that inclusion of studies that deviate from HWE can affect the pooled estimate and be potential sources of heterogeneity across the studies (Trikalinos et al. 2006). Hence, we conducted pooled analysis with and without studies that deviated from HWE.

**Figure 2:**
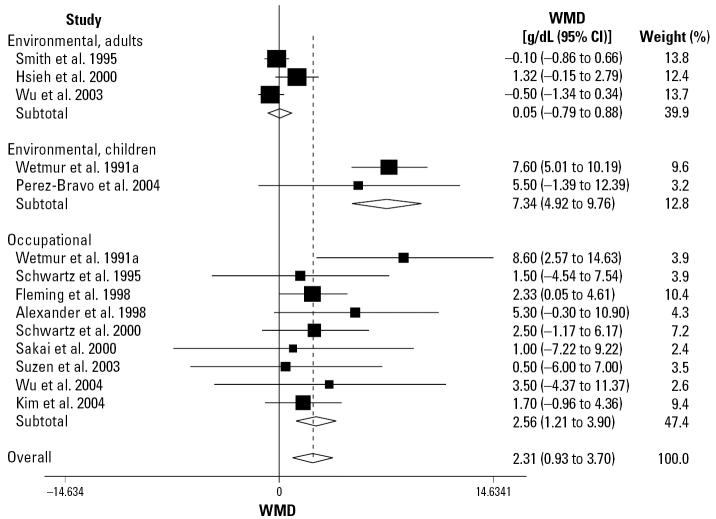
Random-effect WMD and 95% CI in blood lead level between *ALAD1-2/2-2* and *ALAD1-1* carriers.

Pooled WMD analysis among the 14 studies, which included a total of 6,672 subjects, 5,861 (87.84%) were homozygous for *ALAD1* and 811 (12.16%) carried the *ALAD2* allele, showed a large heterogeneity among the studies (χ^2^_13_ = 54.75; *p* = 0.000; *I*^2^ = 76.3%) (Table 3; Figure 2). In subgroup analysis (subgroups were defined by the type of study and by population, that is, occupationally and environmentally exposed adults and children), there was no heterogeneity between occupational studies (*I*^2^ = 0), between the studies of environmentally exposed children (*I*^2^ = 0), and moderate heterogeneity among the studies of environmentally exposed adults (*I*^2^ = 55.2%). After removal of the studies not in HWE, the overall heterogeneity decreased (χ^2^_11_ = 17.92; *p* = 0.07), and the variation in WMD attributable to heterogeneity was moderate (*I*^2^ = 38.6%) (Table 3). Overall, the pooled WMD analysis indicated that the carriers of *ALAD2* allele had a significantly higher BLL (2.31 μg/dL; 95% CI, 0.93 to 3.70) compared with carriers homozygous for the *ALAD1* allele, a finding that was mostly driven by the occupational studies. Removal of the two studies not in HWE resulted in a not significantly higher WMD level of BLL (0.86 μg/dL; 95% CI, −0.1 to 1.73). There was no evidence of publication bias according to Begg’s test (*p* = 1.0, with continuity correction) and Egger’s test (*p* = 0.10).

#### Occupational studies

**Figure 3:**
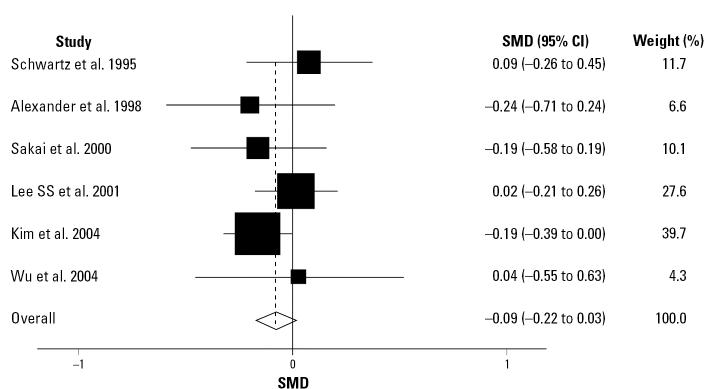
Random-effect SMD in ZPP values between *ALAD1-2/2-2* and *ALAD1-1* carriers.

Lead workers carrying the *ALAD2* allele had higher BLLs (WMD = 2.56 μg/dL; 95% CI, 1.21 to 3.90), with the difference being statistically significant (*p* = 0.027) (Figure 3). Analysis of the studied in HWE (Table 3) resulted in a decreased but still significant higher WMD (2.24 μg/dL; 95% CI, 0.85 to 3.62).

#### Environmental adult studies

By contrast, the WMD in adults environmentally exposed to lead was 0.05 μg/dL (95% CI, −0.79 to 0.88), which was not statistically significant.

#### Environmental children studies

Pooled analysis of the two studies of children showed a WMD in BLL of 7.34 μg/dL (95% CI, 4.92 to 9.76), with the individuals carrying *ALAD2* having significantly higher BLLs (*p* = 0.00). However, the data should be viewed cautiously because other than deviation from HWE, the individuals selected for the study reported by Wetmur et al. (1991a) had higher initial clinical evaluations of elevated erythrocyte protoporphyrin (FEP) levels thus introducing potentially serious selection bias in the study design.

### ALAD *polymorphism and heme synthesis*

#### Zinc protoporphyrin (ZPP)

Six published occupational studies related ZPP to *ALAD* polymorphism (Alexander et al. 1998; Kim et al. 2004; Lee SS et al. 2001; Sakai et al. 2000; Schwartz et al. 1995; Wu et al. 2004). Because the methods used to measure ZPP were not uniform, we calculated the SMD. The overall pooled SMD was −0.09, indicating that individuals carrying the *ALAD 2* allele had lower ZPP values (Figure 3). However, the SMD was not statistically significant (95% CI, −0.22 to 0.03; *p* = 0.13). Heterogeneity was not significant (χ^2^_5_ = 3.88, *p* = 0 .56; *I*^2^ = 0.0%), indicating that the studies were homogeneous. There was no evidence of publication bias according to Begg’s test (*p* =1.0, with continuity correction) and Egger’s test (*p* = 0.37).

#### Hemoglobin

**Figure 4:**
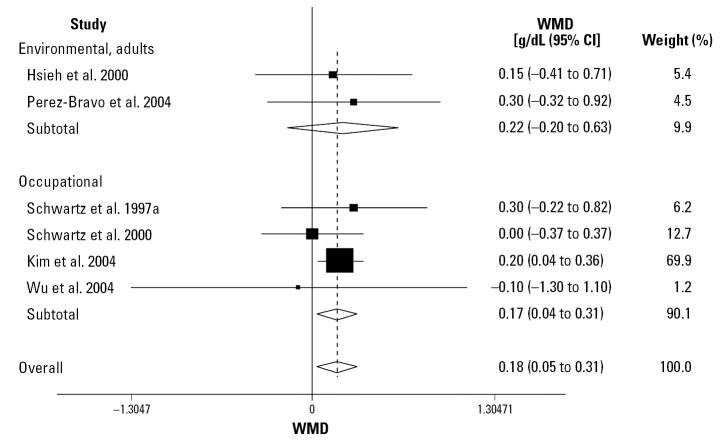
Random-effect WMD in hemoglobin values between *ALAD1-2/2-2* and *ALAD1-1* carriers.

Six published cross-sectional studies related hemoglobin levels to *ALAD* polymorphism: four occupational (Kim et al. 2004; Schwartz et al. 1997a, 2000; Wu et al. 2004), one on environmentally exposed adults (Hsieh et al. 2000), and one on environmentally exposed children (Perez-Bravo et al. 2004) studies. Individuals carrying *ALAD2* had higher hemoglobin measurements (WMD = 0.18 g/dL; 95% CI, 0.05 to 0.31; *p* = 0.007) (Figure 4). However, stratification by study design shows that individuals environmentally exposed to lead carrying ALAD2 had a not statistically significant higher hemoglobin measurement (WMD = 0.22 g/dL; 95% CI, −0.20 to 0.63; *p* = 0.306). Heterogeneity was not significant (χ^2^_5_ = 1.55; *p* = 0.9; *I*^2^ = 0.0%); the studies are thus homogeneous and it is appropriate to use the summary weighted mean. There was no evidence of publication bias according to Begg’s test (*p* = 0.45, with continuity correction) and Egger’s test (*p* = 0.66).

### ALAD *polymorphism and bone compartment*

#### Tibia lead level

Ten studies reported data on lead levels in tibia bone and *ALAD* polymorphism as an outcome measure. Four studies that relied on previous data sets (Lee BK et al. 2001; Lee SS et al. 2001; Weaver et al. 2003; Wu et al. 2003) and two that did not have data based on the polymorphism (Bellinger et al. 1994; Weaver et al. 2003) were excluded, leaving four studies for analysis: two studies involving lead workers (Fleming et al. 1998; Schwartz et al. 2000) and two of environmentally exposed adults (Smith et al. 1995; Wu et al. 2003). Because the methods used to measure tibia lead levels were not the same in all studies, the pooled SMD was calculated. The overall pooled SMD of −0.07 was not significant (95% CI, 0.20 to 0.05) and no significant heterogeneity existed among the studies (*I*^2^ = 0.0%) (Table 4).

#### Trabecular (patella and calcaneus) lead level

Four studies—two occupational studies (Fleming et al. 1998; Theppeang et al. 2004) and two environmental studies (Smith et al. 1995; Wu et al. 2003)—were analyzed for differences in trabecular lead level and *ALAD* polymorphism. The overall pooled SMD of −003 (95% CI: 0.16, 0.09) was not significant and heterogeneity was absent (*I*^2^ = 0.0%) (Table 4).

#### Difference between trabecular and cortical bone lead level

Two environmental studies (Hu et al. 2001; Smith et al. 1995) and one involving lead workers (Fleming et al. 1998) were analyzed for differences between trabecular (patella and calcaneus) and cortical (tibia) bone lead levels and *ALAD* polymorphism. The overall pooled SMD (SMD = 0.03; 95% CI, 0.21 to 0.26) was not significant, but there was moderate heterogeneity (*I*^2^ = 50.9%) (Table 4). Overall, these analyses showed no significant difference between *ALAD* genotypes and trabecular and cortical bone lead concentrations.

### ALAD *polymorphism and DMSA test outcome*

Dimercaptosuccinic acid (DMSA) is a chelating agent used to treat lead intoxication. Two studies (Schwartz et al. 1997b, 2000) reported chelatable urinary lead after administration of oral doses of DMSA. The WMD calculated from these studies showed that individuals homozygous for *ALAD2* had an average of −21.30 μg of DMSA-chelatable lead (95% CI, 40.81 to −1.79; *p* = 0.03) higher than heterozygous workers (Table 4). These data indicate that the bioavailability of lead is greater in *ALAD1-1* individuals than in *ALAD1-2* individuals.

### ALAD *polymorphism and kidney function*

#### Serum creatinine

Four studies reported serum creatinine values and *ALAD* polymorphism (Bergdahl et al. 1997a; Smith et al. 1995; Weaver et al. 2003; Wu et al. 2003). The study by Bergdahl et al. (1997a) was excluded because it was not possible to calculate the mean and SD. Therefore, only three studies were analyzed by pooled SMD: two conducted in environmentally exposed individuals (Smith et al. 1995; Wu et al. 2003), and one in lead-exposed workers (Weaver et al. 2003). Very high heterogeneity was present (*p* < 0.001; *I*^2^ = 92.9% ) (Table 4), that could be attributed to different levels of lead exposure. Pooled analysis of the two studies reporting low levels of lead exposure (environmental studies) shows that individuals carrying the *ALAD2* allele had a corresponding significantly higher serum creatinine (SMD = 0.48; 95% CI, 0.33 to 0.62) than those individuals homozygous for *ALAD1.*

### ALAD *polymorphism and blood pressure*

Two cross-sectional studies related systolic blood pressure to *ALAD* polymorphism (Lee BK et al. 2001; Smith et al. 1995). The pooled WMD was 0.30 mmHg higher in individuals carrying the *ALAD2* allele, but the difference was not statistically significant (95% CI, −2.18 to 2.78) (Table 4).

Heterogeneity was present among three studies (Lee BK et al. 2001; Smith et al. 1995; Wu et al. 2003) relating diastolic blood pressure to *ALAD* polymorphism (χ^2^_2_ = 6.16; *p* = 0.05; *I*^2^ = 66.9%) (Table 4). This heterogeneity was most likely due to the different frequency of the *ALAD2* allele in the population investigated, as well as the level of lead exposure. Exclusion of the occupational study (Lee BK et al. 2001), which has a low frequency of *ALAD2* allele and modestly higher levels of lead exposure, resulted in a nonsignificant test for heterogeneity (χ^2^_1_ = 0.47, *p* = 0.49), a significant pooled WMD that was 1.88 mmHg higher in individuals carrying the *ALAD2* allele (95% CI, 0.46 to 3.31; *p* = 0.01) (Table 4).

## Discussion

Our goal in this study was to determine the associations of *ALAD* polymorphism on blood lead levels and bone deposition, and the role of this polymorphism as a modifier of target organ lead toxicity. Overall, our meta-analysis shows that individuals carrying the *ALAD2* allele had generally higher blood lead levels than those homozygous for *ALAD1*. The data suggest that carrying the *ALAD* allele is a significant determinant for blood lead concentrations among individuals subjected to high levels, such as lead-exposed workers. *ALAD2* does not appear to be a significant determinant of blood lead concentrations among adult individuals exposed to relatively low lead levels (<10 μg/dL).

The biologic plausibility for a differential role of the two *ALAD* alleles lies in the fact that the ALAD2 enzyme could potentially have a higher affinity and stability for lead than ALAD1. Among lead workers, carriers of the *ALAD2* allele had a higher percentage of lead bound to the ALAD enzyme compared to *ALAD1* homozygotes (Bergdahl et al. 1997b). The higher percentage of lead bound to the ALAD 2 enzyme translates to lower levels of bioavailable lead; the reverse is true in *ALAD1* homozygotes. This is consistent with our results. We found that people carrying the *ALAD2* allele had a weighted average of 21.30 μg lower DMSA-chelatable lead than individuals lacking the allele.

The insertion of ferrous iron (Fe^2+^) into the porphyrin ring to form heme is catalyzed by the mitochondrial enzyme ferrochelatase, which shows reduced activity in the presence of lead (Ponka 1997).This reduction in ferrochelatase activity frees protoporphyrin to accept zinc, resulting in the formation of zinc protophorphyrin, which is characteristically increased in both lead poisoning and iron deficiency.

The increased amount of lead bound to the ALAD 2 isozyme should result in decreased lead available to inhibit ferrochelatase, which would thus be available to catalyze the formation of heme with subsequent formation of hemoglobin in the presence of Fe^2+^. In contrast, the weaker binding of lead to ALAD1 results in more bioavailable lead that can inhibit ferrochelatase. This results in increased formation of ZPP and decreased production of heme and hemoglobin.

Our meta-analysis supports these modifying effects of the *ALAD2* allele. Hemoglobin level was 0.18 g/dL (95% CI, 0.05 to 0.31) higher in lead workers with the *ALAD1-2* genotype. Although *ALAD2* carriers had a lower ZPP (SMD = −0.10), the difference was not statistically significant. ZPP is characteristically increased in lead poisoning and starts to rise exponentially only at blood lead concentrations > 30 μg/dL in adults or > 25 μg/dL in children (Baldwin and Marshall 1999). It is thus reasonable to expect a modifying effect on ZPP by *ALAD* polymorphism with increased lead exposure. The absence of a significant effect could be due to differences in exposure levels to the toxicant across the study populations. Schwartz et al. (1995) found that workers carrying the *ALAD2* allele in the plant with the highest lead exposures were associated with lower ZPP measurements. The association of *ALAD2* with lower ZPP was also reported by Alexander et al. (1998), and this association was more pronounced in workers with blood lead concentrations ≥ 40 μg/dL. Significantly higher levels of ZPP were reported in *ALAD1* homozygous Japanese lead workers compared with *ALAD2* carriers at BLLs > 20 μg/dL (Sakai et al. 2000). Overall, these studies indicate that the *ALAD* allele is a modifying factor in the formation of ZPP at higher blood lead levels (> 20 μg/dL), and that *ALAD2* carriers exhibit lower levels of ZPP and higher levels of hemoglobin.

Differences in lead accumulation in various bone types have been reported. Tibia concentrations differ from those observed in the patella. The cortical bone of the tibia represents a long-term storage depot with an elimination half-life for lead in excess of a decade. *ALAD* status may modify the way in which lead partitions between these bone depots (Smith et al. 1995). That is, the variant ALAD2 protein may effectively increase the blood and soft tissue (e.g., spleen and kidney) compartment half-lives of lead, thus decreasing partitioning to the cortical bone compartment. Our meta-analysis did not find a significant association between *ALAD* polymorphism and accumulation of lead in the different bone compartments. More recently, emphasis has focused on the role of vitamin D receptor (*VDR*) polymorphism in modulating the lead level in the bone compartment (Schwartz et al. 2000; Theppeang et al. 2004). The vitamin D endocrine system plays an essential role in calcium homeostasis and bone metabolism. Vitamin D is a prohormone that is metabolically converted to the active metabolite 1,25-dihydroxyvitamin D (calcitriol), which facilitates calcium absorption from the gut and directly stimulates osteoblasts, the bone-forming cells. These effects are mediated through activation of the VDR, which alters the transcription rates of target genes responsible for the biological response (Dusso et al. 2005). Lead is a divalent cation that behaves like calcium in biological systems, and interactions between lead and calcium have been reported. Calcium and calcitriol deficiencies result in increased lead absorption from the gut (Fullmer 1990). Conversely, higher dietary calcium intake results in lower BLLs in children (Mahaffey et al. 1986) and in reduced bone lead accumulation in animals (Bogden et al. 1995). *VDR* polymorphism may thus influence lead uptake and retention in bone storage pools. Theppeang et al. (2004) found a significantly higher patella lead burden in lead workers carrying the *VDR B* allele. Schwartz et al. (2000) previously reported in adjusted analyses that lead workers carrying the *VDR B* allele had significantly higher tibia lead levels (on average 6.4 μg/g) than workers with the *VDR bb* genotype.

Associations of *ALAD* polymorphism and renal effects of lead exposure have also been reported. Smith et al. (1995) found that the *ALAD2* carriers were more susceptible to decrements in renal function as measured by increases in serum creatinine and blood urea nitrogen (BUN). The increased serum creatinine in individuals carrying *ALAD2* was confirmed in a sample of 89 lead workers (Bergdahl et al. 1997a). Conversely, Korean lead workers with the *ALAD1-2* genotype exhibited lower BUN and serum creatinine (Weaver et al. 2003). The pooled SMD in our meta-analysis showed higher serum creatinine values among *ALAD2* carriers. However, there was significant heterogeneity among the studies that might be ascribed to the level of lead exposure, frequency of the polymorphism in the population investigated, and other possible confounders (e.g., age, sex). Pooled analysis of the studies reporting low levels of lead exposure (environmental studies) shows that individuals carrying the *ALAD2* allele had a corresponding statistically significant mean average of 0.10 mg/dL higher serum creatinine than those individuals homozygous for *ALAD1.*

The effect of lead on blood pressure has also been widely investigated (Kopp et al. 1988; Pirkle et al. 1985). The available literature suggests that there is a positive, albeit weak association between systolic blood pressure and blood lead concentration. A recent meta-analysis showed that a 2-fold increase in blood lead concentration is associated with a rise in systolic pressure of 1.0 mmHg (95% CI, 0.5 to 1.4; *p* < 0.001) and an increase in diastolic pressure of 0.6 mmHg (95% CI, 0.4 to 0.8; *p* < 0.001) (Nawrot et al. 2002). Our meta-analysis did not find a difference in systolic blood pressure associated with *ALAD* polymorphism. However, individuals carrying the *ALAD2* allele who were environmentally exposed to lead showed an increase in diastolic blood pressure of 1.88 mmHg.

The biological plausibility of a causal relationship between elevated blood pressure and lead exposure has been studied mainly in animals and *in vitro*. Experiments have demonstrated that lead affects the smooth muscles of blood vessels by interfering with the Na^+^/K^+^-pump, cyclic AMP, calcium ions (Ca^2+^), and the rennin–angiotensin system (McAllister et al. 1971; Roels et al. 1990; Sandstead et al. 1970). In this context, the presence of other polymorphic genes, such as that coding for endothelial nitric oxide synthase (eNOS), may play an additional role. Endothelial NOS converts l-arginine into nitric oxide, causing relaxation of vascular smooth muscle (Vaziri and Ding 2001; Vaziri et al. 1999) and associations among *eNOS* genotypes, hypertension, lead exposure, and intracellular Ca^2+^ concentrations have been reported (Colombo et al. 2002; Sofowora et al. 2001).

## Conclusions

Measurement of blood lead level is the most convenient, readily available, and logistically feasible biomarker for assessing risk of lead toxicity. However, the presence of the *ALAD2* allele may obscure the clinical interpretation of blood lead values in terms of target organ toxicity. *ALAD2* carriers generally show higher BLLs in adults at increased levels of lead exposure, and appear to be protected against adverse hemapoietic effects as measured by hemoglobin levels. The modifying effects of ALAD on other organs remain unclear, partly because of the the small number of studies. These numbers are relatively small and therefore any inferences have to be cautious (Ioannidis et al. 2003). The strength of the present analysis, however, is based on the aggregation of published studies, thus there is more information for investigating the effect of the allele under investigation. Moreover, the role of other genes such as *VDR* could alter lead deposition in bone. The increasing application of molecular epidemiologic methods has emphasized the interaction between genes and the environment. Multiple gene polymorphisms suggest that genes having a small effect may interact to determine the overall risk. This meta-analysis identifies several issues: *a*) there are numerous potential sources of heterogeneity, including varying allele frequencies and HWE in the populations; *b*) in the context of gene-environment interactions, gene–gene interactions may play a role (for example, ALAD, VDR, and eNOS may interact to modify lead levels in several organs).

## Figures and Tables

**Table 1:** Characteristics of the studies included in the review.

Source	Population	Gene	Variables
Ziemsen et al. 1986^a^	Lead workers	*ALAD*	BLL
Astrin et al. 1987^a^	Children	*ALAD*	BLL
Wetmur et al. 1991a^a^	Lead workers and children	*ALAD*	BLL
Smith et al. 1995	Carpenters	*ALAD*	BLL, PBL, SC, SBP, DBP
Schwartz et al. 1995^b^	Lead workers	*ALAD*	BLL, ZPP
Schwartz et al. 1997a^b^	Lead workers	*ALAD*	BLL, DMSA, ZPP
Schwartz et al. 1997b^b^	Lead workers	*ALAD*	BLL, DMSA, ZPP
Sithisarankul et al. 1997^b^	Lead workers	*ALAD*	BLL, ZPP, HB
Alexander et al. 1998	Lead workers	*ALAD*	BLL, ZPP
Fleming et al. 1998	Lead workers	*ALAD*	BLL, TBL, CBL
Hsieh et al. 2000	General population	*ALAD*	BLL, HB
Sakai et al. 2000	Lead workers	*ALAD*	BLL, ZPP
Schwartz et al. 2000^c^	Lead workers	*ALAD, VDR*	BLL, TBL, DMSA, HB
Lee BK et al. 2001^c^	Lead workers	*ALAD, VDR*	BLL, TBL, DMSA, SBP, DBP
Hu et al. 2001^d^	Veterans	*ALAD*	BLL, TBL, PBL, DMSA
Lee SS et al. 2001^c^	Lead workers	*ALAD, VDR*	BLL, TBL, ZPP, HB
Suzen et al. 2003^e^	Lead workers	*ALAD*	BLL
Wu et al. 2003^d^	Veterans	*ALAD*	BLL, TBL, PBL, SC, DBP
Duydu and Suzen 2003^e^	Lead workers	*ALAD*	BLL
Weaver et al. 2003^c^	Lead workers	*ALAD, VDR, eNOS*	BLL, TBL, SC
Kim et al. 2004	Lead workers	*ALAD*	BLL, ZPP, HB
Theppeang et al. 2004	Lead workers	*ALAD, VDR, eNOS*	PBL
Wu et al. 2004	Lead workers	*ALAD*	BLL, ZPP, HB
Perez-Bravo et al. 2004	Children	*ALAD*	BLL, HB

Abbreviations: BLL, blood lead level; BUN, blood urea nitrogen; CBL, calcaneus bone lead; DBP, diastolic blood pressure; DMSA, DMSA-chelatable lead; HB, hemoglobin; PBL, patella bone lead; SBP, systolic blood pressure; SC, serum creatinine; TBL, tibia bone lead; ZPP, zinc protoporphyrin.

a These studies use the same population data.

b These studies use the same population data.

c These studies use the same population data.

d These studies use the same population data.

e These studies use the same population data.

**Table 2:** Frequency of *ALAD* allele and HWE in the studies analyzed.

Source	Total no.	*ALAD1-1*	*ALAD1-2*	*ALAD2-2*	*ALAD1* (*p*)	*ALAD2* (*q*)	HWE
Occupational studies							
Wetmur et al. 1991a	203	161	32	10	0.872	0.128	No
Schwartz et al. 1995	307	273	34	0	0.945	0.055	Yes
Alexander et al. 1998	134	114	20	0	0.925	0.075	Yes
Fleming et al. 1998	382	312	67	3	0.904	0.096	Yes
Sakai et al. 2000	192	161	29	2	0.914	0.086	Yes
Schwartz et al. 2000	795	716	79	0	0.950	0.050	Yes
Suzen et al. 2003	71	50	21	0	0.852	0.148	Yes
Kim et al. 2004	1,219	1,106	113	0	0.954	0.046	Yes
Wu et al. 2004	57	42	15	0	0.868	0.132	Yes
Environmental studies, adults							
Smith et al. 1995	688	592	94	2	0.929	0.071	Yes
Hsieh et al. 2000	660	630	29	1	0.977	0.023	Yes
Wu et al. 2003	709	595	107	7	0.915	0.085	Yes
Environmental studies, children							
Wetmur et al. 1991a	1,278	1,136	129	13	0.939	0.061	No
Perez-Bravo et al. 2004	93	84	8	1	0.946	0.054	Yes

**Table 3:** Summary effect size of blood lead level in *ALAD1-2/2-2* versus *ALAD1-1* carriers.

					Publication bias tests (*p*-value)	
Population and subgroup analysis	No. of studies	WMD [μg/dL (95% CI)]	χ^2^ test *p*-value	*I*^2^ (%)	Begg (corrected)	Egger
ALL	14	2.31 (0.93 to 3.70)*	0.0	76.3	1.0	0.10
ALL in HWE	12	0.86 (−0.01 to 1.73)	0.07	38.6		
Occupational	10	2.56 (1.21 to 3.90)*	0.65	0.0	1.0	0.42
Occupational in HWE	9	2.24 (0.85 to 3.62)*	0.97	0.0		
Environmentally exposed adults	3	0.05 (−0.79 to 0.88)	0.11	55.2	1.0	0.32
Environmentally exposed children	2	7.34 (4.92 to 9.76)*	0.57	0.0	1.0	NA
Environmentally exposed children in HWE	1	5.5 (−1.39 to 12.39)	NA	NA	NA	NA

NA, not applicable.

* Statistically significant, *p* < 0.05.

**Table 4:** Summary effect size between *ALAD1-2/2-2* and *ALAD1-1* carriers on various outcomes.

						Publication bias tests (*p*-value)	
	No. (type) of studies	SMD	95% CI	Heterogeneity χ^2^ test *p*-value	*I*^2^ (%)	Begg (corrected)	Egger
Cortical (tibia) lead	4 (Combined)	−0.07	−0.20 to 0.05	0.59	0.0	1.00	0.64
	2 (Occupational)	−0.07	−0.28 to 0.14	0.23			
	2 (Environmental)	−0.07	−0.26 to 0.11	0.50			
Trabecular lead (patella and calcaneus)	4 (Combined)	−0.03	−0.16 to 0.09	0.60	0.0	0.09	0.03
	2 (Occupational)	−0.03	−0.21 to 0.15	0.68			
	2 (Environmental)	0.01	−0.30 to 0.31	0.19			
Difference trabecular–cortical	3 (Combined)	0.03	−0.21 to 0.26	0.13	50.9	0.30	0.23
	1 (Occupational)	−0.07	−0.33 to 0.19				
	2 (Environmental)	0.14	−0.35 to 0.64				
DMSA-chelatable lead	2 (Occupational)	−21.30^a^	−40.81 to −1.79^a^	0.85	0.0		
Serum creatinine	3 (Combined)	0.23	−0.24 to 0.70	0.00	92.9	1.00	0.38
	1 (Occupational)	−0.27	−0.50 to −0.04				
	2 (Environmental)	0.48	0.33 to 0.62				
Systolic blood pressure	2 (Combined)	0.30^a^	−2.18 to 2.78^a^	0.33	—	1.00	—
	1 (Occupational)	−1.10^a^	−4.84 to 2.64^a^				
	1 (Environmental)	1.40^a^	−1.92 to 4.72^a^				
Diastolic blood pressure	3 (Combined)	0.81^a^	−1.47 to 3.09^a^	0.05	66.9	1.00	0.24
	1 (Occupational)	−2.00^a^	−4.89 to 0.89^a^				
	2 (Environmental)	1.88^a^	0.46 to 3.31^a^				

a WMD.
